# Trends in Financial Affordability of Healthcare Among Tribal Women of Reproductive Age: A Cross-Sectional Study From India

**DOI:** 10.7759/cureus.73463

**Published:** 2024-11-11

**Authors:** Sagaya Joel Leo, Veena Prasad, Shoyaib K Md

**Affiliations:** 1 Medicine, Pinderfields Hospital, Mid Yorkshire Teaching NHS Trust, Wakefield, GBR; 2 General Internal Medicine, Pinderfields Hospital, Mid Yorkshire Teaching NHS Trust, Wakefield, GBR; 3 Community and Family Medicine, All India Institute of Medical Sciences, Mangalagiri, Guntur, IND

**Keywords:** affordability, healthcare, national family health survey, tribal, women

## Abstract

Background: Universal health coverage (UHC) means that all people have access to the full range of quality health services they need, when and where they need them, without financial hardship. UHC is one of the targets of Sustainable Development Goal (SDG) 3 which India is trying to achieve with various initiatives and health programs. Tribal communities form an integral part of India's population. Due to various geographic barriers to access to the location of their settlements, it becomes problematic to provide essential services including healthcare without good expenditure. Moreover, as a result of various disadvantages, employment and subsequently the affordability for tribal groups poses an issue for availing healthcare services or those which are affordable are far away from the usual reach. With this study we would like to track the progress towards SDG 3 for the tribal communities in India.

Objectives: To assess the trends of ability to afford healthcare for self among tribal women in India over five years and to assess the determinants of this affordability among the same population.

Methods: We used the Individual Recode (IR) datasets of Demographic & Health Surveys (DHS) data of Fourth and Fifth round for secondary data analysis. 670,384 and 689,454 cases from National Family Health Survey (NFHS) 4 and 5 were included for analysis. Under "svy" command, design adjusted chi square test was used, followed by binary logistic regression to derive unadjusted and adjusted odds ratio for various determinants.

Results: 6.38% and 6.23% of women belonged to tribal communities during NFHS 4 and NFHS 5 respectively. Only 0.24% and 0.35% had education above secondary education during NFHS 4 and NFHS 5. Majority of the tribal women were married during both surveys and around 0.3% were pregnant during the interviews. Consequently, most of the women were the wife of the head of household. Majority of the tribal women were followers of the Hindu religion and resided in the rural areas of the country. During NFHS 4, the major proportion of women belonged to the East zone and during NFHS 5, they belonged to the Northeast zone of India. For women in the age group of 25 to 29 years the odds of facing difficulty was the highest (aOR: 1.55 during NFHS 4 and 1.88 during NFHS 5). Moreover, those with no education showed highest odds of facing difficulty in arranging money for healthcare for self during both surveys (aOR: 1.69 during NFHS 4 and 1.45 during NFHS 5) when compared with those with higher education. In addition, the odds of facing affordability issues had increased from NFHS 4 to NFHS 5 for poorest tribal women (aOR 6.65 during NFHS 4 to aOR 8.91 during NFHS 5). There has been significant decrease in odds of facing affordability as a barrier among tribal women residing in Northeast zone of India (aOR: 5.01 during NFHS 4 and aOR: 3.45 during NFHS 5). The odds for facing affordability issues for tribal women residing in rural areas remained similar during both surveys.

Conclusion: There has been a slight decrease in the proportion of tribal women facing financial affordability as a barrier to accessing healthcare. Further factors like middle age groups of 25 to 29 years, no education, divorced or separated marital status, and belonging to poorest category of the Wealth Index were significant determinants due to which financial affordability has become a barrier to avail healthcare for self.

## Introduction

Healthcare affordability is the ability of individuals and families to pay for healthcare services without experiencing financial hardship. It includes various types of costs such as out-of-pocket (OOP) expenditures, insurance premiums, co-payments to name a few [1]. India, which is one of the most populous countries, is also home to over 104 million people belonging to various tribes across the country. Tribals constitute around 8.6% of India's population which is a significant proportion when it comes to healthcare. Many of these are underprivileged citizens in many aspects of life - geographically, socio-economically, and politically [2]. Constitutionally, they are categorized as Scheduled Tribes (ST), of which a large fraction are forest dwellers or live in and around the legally protected forest areas in South, Central, and Northeast India. The ST population has poorer access to healthcare, as evidenced by the higher maternal and infant mortality rate, higher rates of both communicable and non-communicable diseases, and malnutrition [3]. In contrast to the prevailing demographic trends in the broader society, the tribal community exhibits a more favorable sex ratio of 990, surpassing the national average of 943. This phenomenon may be attributed to bride price. This customary norm acknowledges the loss of labor to the bride's family upon marriage and recognizes her role in reproducing the workforce for the new family [4]. Despite this, India was ranked second in the total number of global maternal deaths, with around 24000 fatalities in 2020, with the tribal communities bearing more than 50% of these deaths. A study conducted on the tribal women from the Madia-Gond tribe in the Indian state of Maharashtra concluded that inaccessibility of healthcare stemmed from various factors such as poverty and suboptimal healthcare infrastructure, which is, in turn, influenced by other socio-cultural practices such as using harmful fluids to give baths to newborns, delayed breastfeeding or provision of pre-lacteal feeds, the double burden of household and labor work and limited access to education [5]. 

The tribal population of India endures long-standing discrimination and denial of basic human rights due to societal marginalization from land disputes, socio-economic disparities, geographic isolation, and limited access to healthcare. Ninety percent of the tribal population resides in rural areas, making it furthermore challenging to access healthcare due to the suboptimal primary healthcare system, lack of well-trained grassroots healthcare professionals, and lack of awareness among the rural population. The theme of World Health Day for 2018 was Universal Health Coverage (UHC): everyone, everywhere. The government of India's flagship scheme Ayushman Bharat was launched in 2017 to attain the sustainable development goals of UHC. Pradhan Mantri Jan Arogya Yojana (ABPM-JAY), the world's largest health insurance scheme, aims at providing a health cover of INR 500,000 per family per year to secondary and tertiary care hospitalization to over 55 crore beneficiaries belonging to the poor and vulnerable families [6]. Affordability of healthcare becomes a challenge for poor and vulnerable families as they have to spend money from OOP on top of suffering wage loss to seek healthcare. More than 50 million Indian households fall into poverty annually on account of OOP healthcare expenditure. 

The primary objective of this study was to assess the trend in the prevalence of difficulty in obtaining money for healthcare for self among tribal women of reproductive age group. The secondary objective was to assess the factors affecting the trend in financial affordability among tribal women of reproductive age group at Individual, Household and Community levels. A regression analysis was conducted utilizing data derived from the National Family Health Survey (NFHS) 4 (2015-16) and NFHS 5 (2019-21) to assess the extent to which financial constraints have impeded healthcare access for tribal women of reproductive age. Ayushman-Bharat was introduced after NFHS 4 and before NFHS 5; a comparison between the two would also be directed toward the efficacy of this implementation. With the study, a better picture can be drawn towards what factors such as education, financial independence, and access to audio-visual media play more significant roles in the affordability of tribal women of reproductive age groups to access healthcare. 

## Materials and methods

Demographic and Health Survey (DHS) maintains the data for NFSH surveys in recode files. The Individual Recode (IR) dataset contains details of all the women who were interviewed during the survey. These datasets are recoded datasets for ease of analysis and not the raw datasets. We have used the IR dataset to obtain the data on various variables of interest.

It is a cross-sectional comparative study from two National Family Health Survey rounds. During NFHS 4 and NFHS 5, the study participants were included from all states and union territories of India using multistage cluster sampling combined with the population proportion to sampling (PPS) technique. During NFHS 4 and NFHS 5, a two-stage stratified sample was used, with the 2011 census serving as the sampling frame. In rural areas, villages were the primary sampling units (PSUs) that were selected using PPS, and the strata were defined based on the number of households and the percentage of the population belonging to scheduled castes and tribes. In urban areas, census enumeration blocks were selected using PPS based on SC/ST population percentage. Complete household mapping was conducted in selected PSUs, which were segmented into clusters. Random sampling was used to choose clusters, and within each selected cluster, 22 households were randomly selected in the second stage of data collection. In each household, one woman from the eligible age group was selected randomly for an interview.

The dependent variable is a computed binomial variable based on the question, "Getting medical help for self: difficulty in arranging money," with two outcomes: "Not a problem" and "Yes, a problem." The response as not a problem is taken as the reference category. Further, independent variables include age group, which represents the respondent's current age in a class interval of five years. The 45 to 49 years age group was taken as the reference category. "Highest Education Level", which denotes the respondent's highest education level. Further, other independent variables include current marital status, current pregnancy status, relationship to head of household, wealth index, frequency of reading of newspaper or magazine, listening to radio and watching TV and number of living children at the household level. In addition to the above for the community level, the independent variables were type of residency and geographic zone.

The study included 64,144 cases of tribal women out of 699,686 cases from the women’s dataset for NFHS 4 and 67263 cases of tribal women out of 724115 cases from the women’s dataset for NFHS 5. The duration of the study was one month.

Inclusion and exclusion criteria

Those cases in which response to the question, "do you belong to a scheduled tribe" was "yes" were only included in the final analysis. There were no exclusion criteria.

Ethical considerations

Our study involves the usage of secondary data that is available in the public domain and can be accessed with a formal request from DHS. Each author on this had separately obtained authorization from DHS to comply with its rules and regulations for using datasets of NFHS 4 and NFHS 5. This study does not require any clearance from the Institutional Review Board as the ethical committee clearance was obtained by DHS already at the time of the interview and we are only using anonymised recorded secondary data from the datasets available in the public domain.

Data was extracted from IR files, and dependent and independent variables were computed using STATA MP 4 core v18 (StataCorp., College Station, TX, USA). The association was assessed using an adjusted chi-square test. Further, binomial regression analysis was used to derive the unadjusted and adjusted odds ratio. All the analyses were carried out under the subset of the "svy" command that would account for the complex survey sampling design with due consideration for weights, strata, and primary sampling units in the dataset. At the individual level, predictors such as age group, educational status, access to newspapers, radio, and TV, and current pregnancy status were evaluated. At the household level, the current marital status, wealth index, religion (Hindus or non-Hindus), number of living children, and relationship to the head of the family were considered. The type of residence (rural/urban) and the different geographical zones were the predictors assessed at the community level. 

## Results

Women in the reproductive age group (15-49 years) of various socio-economic backgrounds were included in the analysis. In NFHS 4, the dataset of 64,144 cases of tribal women of reproductive age group was included based on the inclusion criteria. Similarly, from NFHS 5, a dataset of 67,263 cases of tribal women was included. 6.38% and 6.22% of women during NFHS 4 and NFHS 5 respectively belonged to tribal groups. Socio-demographic distribution is given in Table 1. 

**Table 1:** Distribution of Tribal Women facing difficulties in arranging money to get medical help for self NFHS: National Family Health Survey

		NFHS 4 (Weighted %)	NFHS 5 (Weighted %)
Age Group in years	45-49	0.65	0.66
	15-19	1.17	1.11
	20-24	1.15	1.07
	25-29	1.09	1.02
	30-34	0.88	0.89
	35-39	0.79	0.8
	40-44	0.65	0.68
	Total	6.38	6.23
Highest Education level	Higher	0.24	0.35
	No education	2.88	2.34
	Primary	0.9	0.87
	Secondary	2.36	2.67
	Total	6.38	6.23
Current marital status	Married	4.69	4.45
	Never Married	1.41	1.45
	Widowed	0.25	0.24
	Divorced	0.02	0.01
	No longer living together	0.01	0.01
	Total	6.38	6.16
Currently Pregnant	No	6.08	5.95
	Yes	0.3	0.27
	Total	6.38	6.22
Relationship to Head of Household	Head	0.35	0.44
	Wife	3.16	2.93
	Daughter	1.43	1.44
	Daughter in law	0.99	1.05
	Others	0.45	0.37
	Total	6.38	6.23
Wealth Index	Richest	0.25	0.21
	Poorest	2.93	3.11
	Poorer	1.71	1.59
	Middle	0.96	0.88
	Richer	0.53	0.44
	Total	6.38	6.23
Frequency of reading a newspaper or magazine	Not at all	5.03	5.03
	Less than once a week	0.63	0.82
	At least once a week	0.45	0.37
	Almost every day	0.27	0
	Total	6.38	6.22
Frequency of listening to Radio	Not at all	5.54	5.57
	Less than once a week	0.34	0.48
	At least once a week	0.34	0.17
	Almost every day	0.16	0
	Total	6.38	6.22
Frequency of watching TV	Not at all	2.51	2.66
	Less than once a week	0.61	0.14
	At least once a week	0.89	2.16
	Almost every day	2.37	0
	Total	6.38	4.96
Religion	Hindu	5.44	5.36
	Non Hindu	0.94	0.86
	Total	6.38	6.22
No. of living children	Zero	1.92	1.96
	1 to 2	2.37	2.39
	3 to 4	1.66	1.54
	More than 4	0.43	0.33
	Total	6.38	6.22
Type of Residency	Urban	0.83	0.7
	Rural	5.55	5.52
	Total	6.38	6.22
Geographic Zone	South	0.76	0.56
	North	0.47	0.67
	North East	0.71	1.44
	Central	1.38	1.73
	East	1.91	1.11
	West	1.15	0.71
	Total	6.38	6.22

In NFHS 4 and 5, women aged 20 to 24 encountered financial obstacles when seeking medical care for themselves at adjusted odds ratios of 1.19 (CI 1.09-1.30) and 1.16 (CI 1.08-1.23), respectively. Simply put, this indicates that a woman aged 25 to 29 during NFHS 4 faced 1.155 higher adjusted odds (CI 1.07-1.24), which is as many challenges as a woman aged 45 to 49 in accessing medical services. The odds ratio of outcomes for the 15 to 19 age group dropped from 1 (CI 0.90-1.11) in NFHS 4 to 0.90 (CI 0.82-0.98) in NFHS 5, indicating an improvement in access to healthcare affordability for these women. The 30 to 34 age group surveyed showed a rise in aOR from 1.04 (CI 0.97-1.10) in NFHS 4 to 1.16 (CI 1.09-1.23) in NFHS 5, indicating growing challenges for this age group. Subsequently, women with no formal education consistently reported higher adjusted odds of difficulty compared to those with higher education (aOR: 1.70, CI 1.52-1.89 in NFHS 4; aOR: 1.45, CI 1.31-1.61 in NFHS 5). In comparison with a woman with higher education, women with secondary, primary, and no education have 1.16, 1.23, and 1.69 times higher adjusted odds of facing difficulty in accessing healthcare due to affordability concerns. That is, a gradient was observed across education levels, with difficulties generally decreasing as education level increased. However, the decrease in aORs from NFHS 4 to NFHS 5 for all education levels suggests a narrowing but persistent education-related disparity. 

In general, single women (unmarried, widowed, divorced, or no longer living together) had higher odds of difficulty in accessing healthcare compared to married women. Among these, in NFHS 4, divorced women faced the highest odds (aOR 1.49 CI 1.18 -1.86) compared to married women, whereas in NFHS 5, the maximum odds were for the women who have never been married (aOR 1.41 CI 1.30 - 1.53). There was a significant improvement in the aOR for divorced women, from 1.49 in NFHS 4 to 1.26 in NFHS 5. These patterns reflect the complexity of marital status and gender dynamics in determining healthcare affordability for tribal women. In a broad aspect, pregnancy was identified not to be a crucial predictor in differences in the affordability of healthcare among tribal women. In NFHS 4, it was slightly easier for pregnant women to procure finances for medical attention (aOR 0.90 CI - 0.85 - 0.97), while in NFHS 5, there was a slight increase in the adjusted odds of obtaining finances for medical care in pregnant women (aOR 1.04 CI 0.96-1.12) 

Surprisingly, in both NFHS 4 and 5, the woman who is the wife of the head of the family had the highest odds of facing difficulties in arranging money for getting medical help for herself (aOR 1.49 CI 1.38 - 1.61 during NFHS 4 and aOR 1.34 CI 1.24 - 1.46) in comparison to when she is the head herself. Relatively, the daughter of the head of the family had the slightest difficulty in affordability during NFHS 5; the daughter had even lesser difficulty in sourcing finances for medical care compared to the head herself (aOR 0.89 CI 0.82 - 0.98). The adjusted odds ratio for other female members of the household (excluding wife, daughter, and daughter-in-law) had significantly improved from 1.38 (CI 1.24 - 1.54) in NFHS 4 to 1.06 (CI 0.95-1.17) in NFHS 5. This highlights the significance of household position in obtaining money for medical help among tribal women. 

Wealth index (WI) is one of the most robust predictors of affordability for tribal women to access medical care for themselves, as expected. In NFHS 4, tribal women of the poorest category of the wealth index have about 6.66 (CI 5.12 - 6.6) times more difficulty compared to the wealthiest quintile. This has furthermore increased in NFHS 5 to a striking adjusted odds ratio of 8.91 (CI 7.57 - 10. 48) among the women of the poorest category. A similar pattern of worsening aOR was noted in all categories of wealth index from NFHS 4 to NFHS 5 except among the richer quintile, where there was a slight improvement from 1.266 (CI 1.03 - 1.55) to 1.24 (1.06 - 1.45). 

In NFHS 5, women who did not read newspapers or magazines at all reported the highest difficulties (5.03%). However, the aOR for those reading at least once a week increased from 0.99 (CI 0.91-1.07) in NFHS 4 to 1.14 (CI 1.05-1.22) in NFHS 5. Interestingly, women who read newspapers almost every day faced higher odds of obtaining money for themselves for medical care in comparison to women who do not read newspapers at all (aOR 1.13 CI 1.01-1.25). Moreover, in NFHS 4, tribal women who listen to the radio not at all or less than once a week had higher odds of accessing healthcare compared to women who listen to radio at least once a week or almost every day. Although there was a slight decrease in the adjusted odds ratio (aOR 1.11 CI 1.00-1.23 in NFHS 4 and aOR 1.00 CI 0.94-1.07) among women who listen to radio less than once a week and a slight increase in those among the women who listen at least a week (NFHS 4: aOR 0.96 CI 0.89-1.04; NFHS 5: aOR 0.99 CI 0.89-1.01), these changes or differences are not a significantly high number compared to the other predictors assessed in the study. In addition to the above, in both NFHS 4 and 5, the difficulties faced by tribal women in sourcing money to access healthcare for themselves were higher compared to those of women who did not watch TV at all. However, when comparing NFHS 4 and NFHS 5, there has been an improvement in the adjusted odds ratio in all categories, suggesting a general decrease in difficulties among tribal women in arranging money for healthcare. For instance, the aOR of women who watched TV at least once a week in NFHS 4 was 1.33 (CI 1.24 - 1.43) times harder compared to women who do not watch TV at all. In NFHS 5, this improved to only 1.08 (CI 1.30 - 1.13) times difficulty in the same category of watching TV at least once a week. 

The disparity based on religion has slightly improved from NFHS 4 (aOR among non-Hindus compared to Hindus: 1.25 CI 1.15-1.34) to NFHS 5 (aOR 1.16 CI 1.08-1.25). Moreover, women with more children generally reported lower odds of difficulty. In NFHS 5, compared to women with no children, those with one to two children had an aOR of 0.76 (95% CI: 0.72-0.81), those with three to four children had an aOR of 0.71 (95% CI: 0.67-0.76) and those with more than four children significantly low difficulty in affordability as evidenced by aOR of 0.61 (CI 0.56-0.66). 

Further, rural residents consistently reported higher challenges (5.55% in NFHS 4, 5.52% in NFHS 5) compared to their urban counterparts (0.83% in NFHS 4, 0.7% in NFHS 5) (aOR: 1.53, 95% CI: 1.36-1.74 in NFHS 4; aOR: 1.52, 95% CI: 1.37-1.70 in NFHS 5). There has been nearly no improvement in the odds ratio for tribal women in rural areas between NFHS 4 and 5. Tribal women in the Northeast region faced considerably higher odds of difficulty (aOR: 5.01, 95% CI: 4.22-5.95 in NFHS 4; aOR: 3.92, 95% CI: 3.46-4.45 in NFHS 5) compared to the South. The West region also showed high odds of difficulty (aOR: 2.69, 95% CI: 2.23-3.25 in NFHS 4; aOR: 2.69, 95% CI: 2.35-3.08 in NFHS 5). Meanwhile, women residing in the East and Central zones had relatively less difficulty in obtaining money for healthcare for themselves compared to women from the South zone. 

Table 2 has the details of the adjusted odds ratio of facing difficulties in arranging money for getting medical help for self among tribal women in both NFHS 4 and 5. 

**Table 2:** Adjusted Odds Ratio of facing difficulties in arranging money for getting medical help for self among Tribal Women NFHS: National Family Health Survey

		NFHS 4	NFHS 5
Age Group in years	45-49	1 (-)	1 (-)
	15-19	1.00387 (0.9073061-1.11071)	0.9028928 (0.8282313-0.9842847)
	20-24	1.193528 (1.094334-1.301713)	1.157092 (1.080446-1.239176)
	25-29	1.15512 (1.072822-1.24373)	1.188596 (1.118766-1.262785)
	30-34	1.035514 (0.9720997-1.103065)	1.15974 (1.092816-1.230761)
	35-39	0.9567928 (0.8952497-1.022567)	1.031606 (0.9771835-1.08906)
	40-44	0.9381626 (0.8818549-0.9980656)	1.084336 (1.026139-1.145833)
Highest Education level	Higher	1 (-)	1 (-)
	No education	1.696081 (1.521322-1.890915)	1.453354 (1.313797-1.607735)
	Primary	1.235977 (1.109524-1.376841)	1.104319 (1.000638-1.218742)
	Secondary	1.164497 (1.05914-1.280334)	1.066667 (0.9791902-1.161959)
Current marital status	Married	1 (-)	1 (-)
	Never Married	1.320981 (1.214972-1.436239)	1.412369 (1.300102-1.534329)
	Widowed	1.32814 (1.208827-1.459229)	1.303828 (1.183018-1.436975)
	Divorced	1.483904 (1.183392-1.860729)	1.257594 (1.008367-1.568419)
	No longer living together	1.284996 (1.107417-1.49105)	1.358588 (1.183985-1.55894)
Currently Pregnant	No	1 (-)	1 (-)
	Yes	0.9074066 (0.8478195-0.9711818)	1.041337 (0.9605804-1.128884)
Relationship to Head of Household	Head	1 (-)	(-)
	Wife	1.492273 (1.380859-1.612675)	1.344999 (1.235914-1.463712)
	Daughter	1.115201 (1.020072-1.219202)	0.8979294 (0.8205857-0.982563)
	Daughter in law	1.363039 (1.251331-1.484719)	1.286509 (1.176179-1.407189)
	Others	1.385307 (1.242531-1.544489)	1.060851 (0.9590102-1.173507)
Wealth Index	Richest	1 (-)	(-)
	Poorest	6.659333 (5.120869-8.659998)	8.910822 (7.573846-10.48381)
	Poorer	3.070079 (2.401505-3.924781)	3.633873 (3.085722-4.279397)
	Middle	1.835606 (1.443636-2.334)	2.077009 (1.759885-2.451277)
	Richer	1.266174 (1.032792-1.552293)	1.241969 (1.06098-1.453833)
Frequency of reading a newspaper or magazine	Not at all	1 (-)	1 (-)
	Less than once a week	0.913144 (0.8535876-0.9768557)	1.060068 (1.006612-1.116362)
	At least once a week	0.9871224 (0.9101988-1.070547)	1.135171 (1.053148-1.223581)
	Almost every day	1.126247 (1.015798-1.248706)	- (---)
Frequency of listening to Radio	Not at all	1 (-)	1 (-)
	Less than once a week	1.113605 (1.003628-1.235632)	1.005513 (0.9437946-1.071268)
	At least once a week	0.9699602 (0.8987242-1.046843)	0.9938062 (0.8964757-1.101704)
	Almost every day	0.9723879 (0.8654477-1.092542)	- (---)
Frequency of watching TV	Not at all	1 (-)	1 (-)
	Less than once a week	1.134373 (1.055884-1.218697)	1.063188 (1.013488-1.115325)
	At least once a week	1.332854 (1.241642-1.430766)	1.082471 (1.030421-1.13715)
	Almost every day	1.154179 (1.053678-1.264266)	- (---)
Religion	Hindu	1 (-)	1 (-)
	Non Hindu	1.247815 (1.154601-1.348554)	1.168305 (1.088508-1.253953)
No. of living children	Zero	1 (-)	1 (-)
	1 to 2	0.906147 (0.8542407-0.9612073)	0.7633263 (0.722533-0.8064229)
	3 to 4	0.838006 (0.7818303-0.8982179)	0.7125701 (0.6689209-0.7590675)
	More than 4	0.7370399 (0.6720856-0.8082719)	0.6118781 (0.5625999-0.6654726)
Type of Residency	Urban	1 (-)	1 (-)
	Rural	1.534455 (1.355757-1.736706)	1.522889 (1.367458-1.695986)
Geographic Zone	South	1 (-)	1 (-)
	North	1.248804 (1.02999-1.514102)	1.370379 (1.191429-1.576206)
	North East	5.010891 (4.219271-5.951035)	3.922086 (3.456915-4.449851)
	Central	0.9876001 (0.8303469-1.174634)	0.7965496 (0.7070592-0.8973665)
	East	0.9635881 (0.8092116-1.147416)	0.7889191 (0.6963189-0.8938338)
	West	2.694397 (2.231525-3.253282)	2.690402 (2.349772-3.080411)

## Discussion

This study examines the factors and their trend that are associated with the affordability of healthcare services for the women of reproductive age group in tribal areas of India by comparing data from two national surveys, i.e., NFHS-4 (2015-16) and NFHS-5 (2019-21). The out-of-pocket expenditure (OOPE) in India is one of the highest in the world [7]. The results of this study indicate that affordability of healthcare remains a significant challenge for tribal women, with uncertain degrees of difficulty across individual, household, and community-level predictors. Women aged 24-29 consistently faced the most difficulty in affording healthcare in both surveys, while younger women between 15 and 19 showed improvements between the NFHS 4 and 5 surveys, which could be attributed to targeted government programmes like adolescent health initiatives or schemes aimed at improving maternal and child health services among younger women. Education played a key role, as women with no formal education reported higher odds of difficulty compared to those with higher education. However, the disparity decreased between the two surveys. 

Expectedly, the wealth index occurred as the strongest predictor for the affordability of healthcare. The extensive range in CI shows the higher gravity of the situation, suggesting that the number could be much worse in specific sub-categories of women among the poorest ones. A clear gradient was observed across wealth quintiles, with the odds of difficulty decreasing as wealth increased. An increase in aORs for all wealth quintiles indicates widening economic disparities in healthcare access. These findings underscore the critical role of economic status in healthcare access among tribal women. The percentage of scheduled tribes below the poverty line is very high when compared to the other communities of the population in India [8]. Women from the poorest households faced significantly higher difficulties, with an increase in disparity between NFHS 4 and 5. Similar findings were seen in the study done in tribal people, where 11% of the population surveyed could not afford healthcare services because of inability to pay [9]. The National Health Mission (NHM) in India has been trying for a very long time to reduce the OOPE, specifically among the deprived, disadvantaged, and most vulnerable groups, but in vain due to the proportions of private institutions (around 70%) in the Indian health system [10,11]. The introduction of Ayushman Bharat and the PM-JAY scheme between NFHS 4 and NFHS 5 were aimed at easing the financial barriers, but this study reveals limited progress in some key areas, particularly for women from the poorest wealth group. Further, there has been a slight improvement in disparity of being able to financially afford healthcare among women based on religion, hopefully showing better health coverage for women across all religions among tribal women.

Women from the Northeast and West zones faced the highest odds of financial difficulty in affording healthcare, while the situation is slightly better in the Northeast from NFHS 4 to 5. The findings of our study coincide with the study by Meher et al. (2007), who also found that tribal populations had difficulties in affording and accessing healthcare due to poverty, geographic isolation, and lack of education [12]. The decrease in the aOR for the Northeast region is a satisfactory drop, highlighting better healthcare access provision for tribal women of the Northeast. However, in the west zone, it is consistently high, meaning modifications must be placed in the existing healthcare position in this zone. 

Unsurprisingly, women from rural areas continue to experience more significant challenges than women in urban areas, suggesting that healthcare in rural areas still needs to be developed despite efforts for Universal Health Coverage. The rural-urban divide in healthcare affordability, as observed in this study, is also consistent with findings by Kumar et al. (2018), who highlighted the inadequate healthcare infrastructure in rural India as a critical barrier to accessing healthcare [13]. Based on our study, roughly the wife is followed by other female members of the family, then the daughter-in-law, the daughter, and then the head herself faces the highest to lowest difficulty in the hierarchy. The results highlight the vital need for further planning to improve healthcare facilities for the rural population of tribal women through universal health coverage. These rural-urban disparities underscore the need for tailored, region-specific approaches to improving healthcare affordability for tribal women. Other similar studies also showed that healthcare utilization is higher among the urban population than the rural population [14,15]. Also, it should be noted that frequent exposure to newspapers or TV did not consistently lead to better situations, suggesting a complex connection between media consumption and healthcare affordability. The general picture paints an image of non-Hindus women of the tribal community facing greater difficulties in accessing money for healthcare compared to Hindus. The results suggest that despite the introduction of government schemes like Ayushman Bharat, healthcare affordability among tribal women, especially in certain age groups, education levels, and geographic regions, continues to be a significant concern. 

A significant strength of this study is the large and representative sample size from both NFHS 4 and NFHS 5 surveys, confirming strong findings. The use of 13 predictors across individual, household, and community levels provided a comprehensive understanding of the factors affecting healthcare affordability among tribal women. Additionally, the comparison between NFHS 4 and NFHS 5 offers valuable insights into the effectiveness of recent healthcare policies, such as Ayushman Bharat, on reducing healthcare affordability hurdles for tribal women. The study's multi-level analysis also highlights the interaction of socio-economic, demographic, and geographic factors, enabling a better understanding of the challenges faced by tribal women in accessing healthcare. Moreover, the sample of women in our study comes from the surveys that were carried out on the national level to ensure that health-related data can be obtained from a nationally representative population. Hence, the findings of our study are applicable at the national level and can be used as a first step towards policy-making and implementation for the tribal groups in India. Despite its strengths, the study has its own limitations. First, the use of secondary data from NFHS limits the analysis to pre-existing variables, excluding some potentially essential factors such as specific health-seeking behaviors, use of traditional medicine or other practices, or the impact of non-governmental interventions, given loads of private institutions across the country, as mentioned earlier. Additionally, while the study examines affordability, it does not explore other critical dimensions of healthcare access, such as quality of care or availability of services, which may be equally crucial for improving healthcare outcomes. The reliance on these self-reported data, particularly for socio-economic indicators, may also introduce some bias, as they are subjective and not objective. Respondents may under-report or over-report their income, health expenses, or media consumption, which could affect the accuracy of the results. Generally, NFHS is completed within a year, but NFHS 5 took three years to complete, i.e., from 2019 to 2021. The duration of the survey was prolonged due to the COVID-19 outbreak and lockdown. However, the pandemic has not significantly affected the data for this study. 

The findings of this study are particular to the tribal population in India and may not be generalizable to the broader population or tribal communities in other countries. The unique socio-economic, cultural, and geographic conditions faced by India's tribal women of reproductive age create distinct challenges in healthcare affordability that differ from other disadvantaged groups globally. However, the trends observed in relation to wealth disparities, education, and rural-urban divides could offer insights into similar populations in other developing countries, particularly those with significant rural and indigenous populations. 

## Conclusions

The overall proportion of tribal women who have been facing difficulty in arranging money for getting healthcare for self has been slightly reduced from NFHS 4 to NFHS 5. However, those disadvantaged by virtue of various non-modifiable factors have become more prone to face higher odds of affordability as a barrier to obtain healthcare. Some of the factors at individual level that were posing a higher risk for affordability are age group of 20 to 24 years, no formal education of these tribal women, non-exposure to mass media and non-pregnant state. At the household level, marital status other than married, belonging to poorest category of wealth index, and some of the relationships to the head of the household apart from wife. Further, type of rural residency had been impacting in a similar way among both surveys at the community level. In addition, belonging to Northeast zone has emerged as the area with highest odds of facing affordability as a barrier for getting healthcare for self among these tribal women.

Our study can serve as a starting point towards better policy-making and implementation which is comprehensive and tailor-made for tribal women in order to progress towards Universal Health Coverage with higher efficiency and inclusivity. Our study has highlighted various disparities in financial affordability. Policymakers can take the findings and proceed with further research to explore other important factors that can hinder or favour faster achievement of Universal Health Coverage in India.

## References

[1] Zavras D (2020). Studying healthcare affordability during an economic recession: the case of Greece. Int J Environ Res Public Health.

[2] (2024). Report of the Expert Committee on Tribal Health: Tribal health in India, bridging the gap, a roadmap for the future, executive summary and recommendations. https://nhm.gov.in/index1.php.

[3] N Srinivas P, Seshadri T, Velho N (2023). Response to correspondence article on the research protocol titled Towards Health Equity and Transformative Action on tribal health (THETA) studyto describe, explain and act on tribal health inequities in India: a health systems research study protocol. Wellcome Open Res.

[4] Mutatkar RK (2022). Tribal health issues: need of tribal health policy. Indian J Med Res.

[5] Madankar M, Kakade N, Basa L, Sabri B (2024). Exploring maternal and child health among tribal communities in India: a life course perspective. Glob J Health Sci.

[6] Zodpey S, Farooqui HH (2018). Universal health coverage in India: progress achieved & the way forward. Indian J Med Res.

[7] Goli S, Rammohan A, Moradhvaj Moradhvaj (2018). Out-of-pocket expenditure on maternity care for hospital births in Uttar Pradesh, India. Health Econ Rev.

[8] Ashifa KM (2022). Situation analysis of the social well-being of elderly during the COVID-19 pandemic. Int J Health Sci.

[9] Sumirtha G, Veenapani RV, Umakant D (2017). Health seeking behavior among particularly vulnerable tribal groups: a case study of Nilgiris. J Public Health Epidemiol.

[10] Mohanty SK, Mishra RS, Mishra S, Sen S (2020). Understanding equity of institutional delivery in public health centre by level of care in India: an assessment using benefit incidence analysis. Int J Equity Health.

[11] Manna S, Singh D, Ghosal S, Rehman T, Kanungo S, Pati S (2023). Out-of-pocket expenditure and its correlates for institutional deliveries in private and public healthcare sectors in India: findings from NFHS 5. BMC Public Health.

[12] Meher R (2007). Livelihood, poverty and morbidity. J Health Manag.

[13] Kumar S, Sharma R (2018). Key barriers in the growth of rural health care: an ISM-MICMAC approach. Benchmarking Int J.

[14] Stephenson R, Matthews Z (2004). Maternal healthcare service use among rural-urban migrants in Mumbai, India. Asia Pac Popul J.

[15] Fotso JC, Ezeh A, Oronje R (2008). Provision and use of maternal health services among urban poor women in Kenya: what do we know and what can we do?. J Urban Health.

